# Cryptococcus laurentii Bloodstream Infection in an Immunocompetent Adult: A Rare Central Line-Associated Infection Complicating Extended-Spectrum Beta-Lactamase Klebsiella pneumoniae Bacteremia

**DOI:** 10.7759/cureus.110473

**Published:** 2026-06-08

**Authors:** Khaled Abu Baker, Osama Obeidat, Saphra Sohail, Marie Kima

**Affiliations:** 1 Internal Medicine, HCA Florida North Florida Hospital, Gainesville, USA; 2 Infectious Diseases, HCA Florida North Florida Hospital, Gainesville, USA

**Keywords:** central line-associated bloodstream infections (clabsi), central line-associated infections (clabsi), cryptococcus laurentii, extended-spectrum β-lactamase (esbl), post-operative osteomyelitis

## Abstract

*Cryptococcus laurentii* (*Papiliotrema laurentii*) is a rare opportunistic yeast traditionally considered non-pathogenic in humans. We present a case of a 54-year-old immunocompetent male with a history of osteomyelitis and an indwelling peripherally inserted central catheter (PICC) who developed bloodstream infection with extended-spectrum beta-lactamase (ESBL)-producing *Klebsiella pneumoniae*, followed by catheter-associated fungemia with *C. laurentii*. The patient had no history of diabetes mellitus, malignancy, organ transplantation, or immunosuppressive therapy, and HIV testing was negative. He had been receiving intravenous vancomycin at a rehabilitation facility and admitted to tampering with his catheter, including showering without protection. Upon hospital transfer, empiric therapy with piperacillin-tazobactam and vancomycin was initiated. Blood cultures grew ESBL-producing *K. pneumoniae*, prompting a switch to meropenem. Transthoracic echocardiography showed no vegetations. Subsequent blood cultures grew *C. laurentii*. The PICC line was removed upon confirmation of fungal growth. Lumbar puncture revealed clear cerebrospinal fluid with normal cell counts, glucose, protein, and negative cultures, excluding central nervous system involvement. Fluconazole 400 mg daily was initiated intravenously and transitioned to oral therapy. The patient completed a total of two weeks of fluconazole and meropenem, with documented blood-culture clearance prior to discharge. This case highlights the emerging pathogenicity of *C. laurentii* in immunocompetent hosts with indwelling catheters and emphasizes the importance of catheter hygiene and early fungal identification in catheter-associated bloodstream infections.

## Introduction

*Cryptococcus laurentii*, recently reclassified as *Papiliotrema laurentii*, is a rare opportunistic yeast historically regarded as a saprophyte with limited pathogenic potential in humans [1]. Non-neoformans cryptococci are basidiomycete (encapsulated) yeasts prevalent worldwide, identified from various environmental sources including air, soil, water, pigeon droppings, and food products [2]. *Cryptococcus* species can colonize humans through the respiratory and gastrointestinal tracts, and in patients with impaired cellular immunity, they can become opportunistic pathogens [2]. *C. laurentii* and *C. albidus *together account for approximately 80% of non-neoformans cryptococcal infections [1-3].

Although non-neoformans cryptococcal infections have been reported predominantly in immunocompromised hosts - particularly those with advanced HIV infection and transplant recipients - increasing reports have documented infections in immunocompetent individuals [2-4]. Indwelling intravascular catheters and nosocomial acquisition have been identified as the most common risk factors for non-neoformans cryptococcal fungemia [3-5]. A systematic review by Khawcharoenporn et al. found that the presence of invasive devices was the most significant risk factor specifically for *C. laurentii* infection (adjusted OR = 8.7; 95% CI = 1.48-82.9; p = 0.003) [3]. However, a large retrospective series from the Mayo Clinic by Cano et al. found that among 54 patients with cultures growing *Cryptococcus* species other than *C. neoformans* and *C. gattii*, only 15% of cases were considered potentially pathogenic, with only one case of invasive disease, underscoring the importance of distinguishing true infection from colonization [6].

Despite its rarity, *C. laurentii* bloodstream infection in immunocompetent patients with central venous catheters represents an emerging clinical entity that warrants attention. We present a unique case of *C. laurentii* catheter-associated fungemia complicating extended-spectrum beta-lactamase (ESBL)-producing *Klebsiella pneumoniae* bacteremia in an immunocompetent adult - a dual-pathogen scenario not previously described in the literature.

## Case presentation

A 54-year-old man with a history of emphysema, anxiety, and chronic osteomyelitis following a right tibial fracture was transferred from a rehabilitation facility due to sepsis. He had been receiving intravenous vancomycin via a peripherally inserted central catheter (PICC) for treatment of osteomyelitis and was admitted to showering without covering the line. His medical history was notable for the absence of diabetes mellitus, malignancy, organ transplantation, chronic kidney or liver disease, or prior immunosuppressive or corticosteroid therapy. He had no history of HIV risk factors.

On arrival, he was tachycardic (heart rate 112 beats per minute) and hypoxemic (oxygen saturation 88% on room air), requiring high-flow nasal cannula. Laboratory results showed elevated troponin (669 → 352 → 952 ng/L), and electrocardiography demonstrated diffuse ST-T changes. Transthoracic echocardiography revealed preserved ejection fraction (60-65%), mild diastolic dysfunction, and no vegetations. CT angiography excluded pulmonary embolism, and left-heart catheterization revealed mild coronary calcification with patent vessels. HIV antigen/antibody testing was negative, and the absolute neutrophil count was within normal limits.

Empiric therapy with piperacillin-tazobactam and vancomycin was initiated. Two sets of blood cultures (two aerobic and two anaerobic bottles) drawn on admission grew ESBL-producing *K. pneumoniae*, and treatment was switched to meropenem 1 g intravenously every eight hours, consistent with current Infectious Diseases Society of America (IDSA) guidance recommending carbapenems as preferred therapy for ESBL-producing Enterobacterales bloodstream infections [7]. This recommendation is supported by the MERINO trial, which demonstrated 30-day mortality of 12.3% with piperacillin-tazobactam versus 3.7% with meropenem in patients with ceftriaxone-nonsusceptible *Escherichia coli* or *K. pneumoniae* bloodstream infections [8].

Subsequent blood cultures obtained on hospital day 3 grew yeast, identified as *C. laurentii* by the VITEK 2 method. Catheter-tip culture was not performed. The PICC line was removed upon confirmation of fungal growth, as catheter removal is a key component of management for catheter-related fungemia [3-5]. Fluconazole 400 mg daily was initiated. Antifungal susceptibility testing was performed using the Clinical and Laboratory Standards Institute (CLSI) broth microdilution method and demonstrated a fluconazole minimum inhibitory concentration (MIC) of 2 µg/mL, indicating susceptibility and supporting the use of fluconazole monotherapy.

Lumbar puncture was performed to rule out cryptococcal meningitis, as recommended by the European Confederation of Medical Mycology (ECMM), the International Society for Human and Animal Mycology (ISHAM), and the American Society for Microbiology (ASM) global guideline for all patients with suspected or confirmed cryptococcosis [1]. The cerebrospinal fluid (CSF) results are summarized in Table 1, effectively excluding central nervous system (CNS) involvement. MRI of the right leg showed no active osteomyelitis. Transesophageal echocardiography showed no vegetations, excluding endocarditis.

**Table 1:** Cerebrospinal Fluid Analysis Results Demonstrating the Absence of Central Nervous System Involvement

Parameter	Result	Reference Range
Appearance	Clear, colorless	Clear, colorless
WBC count	1 cell/µL	0-5 cells/µL
RBC count	0 cells/µL	0 cells/µL
Glucose	62 mg/dL	40-70 mg/dL
Protein	32 mg/dL	15-45 mg/dL
Gram stain	No organisms seen	No organisms
Fungal culture	No growth	No growth
Cryptococcal antigen	Negative	Negative
Opening pressure	14 cm H_2_O	6-20 cm H_2_O

Repeat blood cultures obtained 48 hours after catheter removal showed no growth, confirming microbiological clearance. The patient completed a total of two weeks of meropenem and two weeks of fluconazole 400 mg daily following catheter removal. The rationale for fluconazole monotherapy was based on clinical stability, confirmed fluconazole susceptibility, successful catheter removal as source control, confirmed absence of CNS and endovascular involvement, and documented blood culture clearance. The patient was discharged in stable condition with clinical and microbiological resolution.

## Discussion

This case demonstrates that *C. laurentii* can cause clinically significant catheter-associated fungemia even in immunocompetent individuals. The infection's likely source was catheter contamination, compounded by the patient's poor catheter hygiene and exposure to water. Indwelling intravascular catheters and nosocomial acquisition have been identified as the most common risk factors for non-neoformans cryptococcal fungemia [3]. A systematic review by Khawcharoenporn et al. found that the presence of invasive devices was the most significant risk factor specifically for *C. laurentii* infection (adjusted OR = 8.7; 95% CI = 1.48-82.9; p = 0.003) [3].

While *C. neoformans* and *C. gattii* are typically detected by cryptococcal antigen (CrAg) testing via lateral flow assay, *C. laurentii* and other non-neoformans species often require culture-based identification, highlighting a diagnostic limitation [1-2]. The ECMM/ISHAM/ASM global guideline emphasizes that *Cryptococcus* species other than *C. neoformans* and *C. gattii* are rarely pathogenic, and careful assessment of laboratory identification and clinical context is required to ascertain clinical significance (AIII) [1].

Dual infection with ESBL-producing *K. pneumoniae* and *C. laurentii* further complicated management. Carbapenems remain the treatment of choice for serious ESBL-producing Enterobacterales infections, as demonstrated in the MERINO trial and supported by IDSA guidance [8]. Catheter removal and targeted antifungal therapy were pivotal for resolution of the fungemia. The rationale for fluconazole monotherapy was supported by clinical stability, confirmed fluconazole susceptibility (MIC 2 µg/mL by CLSI broth microdilution), successful catheter removal as source control, confirmed absence of CNS and endovascular involvement, and documented microbiological clearance on repeat blood cultures 48 hours after catheter removal. Notably, fluconazole and flucytosine have been reported to have variable activity against non-*neoformans* cryptococci, and fluconazole resistance is more frequent in patients with previous azole exposure [2,9]. Most clinical isolates remain susceptible to amphotericin B [2,10]. Antifungal susceptibility testing is therefore recommended to guide therapy, as elevated MICs against fluconazole and other azoles have been documented in some isolates [1-2]. Echinocandins have no clinical activity against *Cryptococcus* species and are not recommended [2].

## Conclusions

*C. laurentii* should be recognized as a potential pathogen in immunocompetent patients with catheter-associated fungemia and indwelling central venous access devices. Early detection using definitive identification methods, prompt catheter removal, and appropriate antifungal therapy guided by susceptibility testing are essential for favorable outcomes. Clinicians should maintain a high index of suspicion for non-neoformans *Cryptococcus* species in cases of unexplained catheter-associated fungemia, particularly in patients with central venous access devices, even in the absence of traditional immunocompromising conditions.
